# Shared Microbial Blueprints Underlying Symbiotic Plasticity in Desert Plant Endophytes

**DOI:** 10.3390/microorganisms14040836

**Published:** 2026-04-07

**Authors:** Walaa K. Mousa, Ruqaia AlShami, Rose Ghemrawi

**Affiliations:** 1 Department of Pharmacognosy, Faculty of Pharmacy, Mansoura University, Mansoura 35516, Egypt; 2 College of Pharmacy, Al Ain University, Abu Dhabi 64141, United Arab Emirates

**Keywords:** desert plant microbiome, endophytic bacteria, functional convergence, symbiotic plasticity, arid ecosystem adaptation

## Abstract

The desert ecosystem harbors a resilient microbial community that sustains plant life under extreme stress. Understanding the endophytic microbiota of desert flora provides key insights into how these microorganisms enable plant survival and maintain ecological balance in arid landscapes. To date, the endophytic bacterial communities of dominant desert plants in the Arabian Peninsula have not been comprehensively characterized. Here, we investigated the endophytic microbiota of five co-adapted desert species, namely, *Schweinfurthia papilionacea*, *Sesuvium verrucosum*, *Ochtocloa compressa*, *Helianthemum nummularium*, and *Convolvulus arvensis*. These plants coexist in hyper-arid habitats and exhibit exceptional tolerance to drought, salinity, and nutrient scarcity. We hypothesized that, despite their phylogenetic divergence, these plants host functionally convergent microbial communities shaped by desert selection pressures. Using 16S rRNA gene amplicon sequencing, we obtained 3.4 million high-quality reads from 25 samples. Clustering at 97% similarity revealed 35 phyla and 17 dominant genera, highlighting notable microbial richness and ecological complexity. Alpha-diversity indices showed comparable species richness across hosts, while beta-diversity indicated community differentiation driven by environmental filtering. The dominant phyla included *Pseudomonadota*, *Actinomycetota*, *Cyanobacteriota*, and *Bacillota*, reflecting microbial adaptation to extreme desert conditions. Functional pathway prediction revealed enrichment of genes associated with DNA repair and protein turnover, suggesting metabolic flexibility and enhanced survival under stress. Overall, this study provides a comparative metagenomic insight into the endophytic bacterial communities of five desert plant species, uncovering a consistent pattern of functional convergence across diverse hosts. The findings suggest the presence of shared functional traits among the endophytic microbiota examined here, offering preliminary evidence for microbial contributions to plant resilience in arid environments.

## 1. Introduction

Desert plants are living systems of resilience, transforming barren landscapes into self-sustaining ecological niches. Their survival in the absence of stable water and nutrients is not merely a product of plant physiology but a reflection of symbioses with microorganisms that occupy their inner tissues. These endophytic communities play critical roles in modulating stress tolerance, nutrient exchange, and defense mechanisms, effectively extending the plant’s metabolic and ecological capacity [1]. Multiple reviews have highlighted how endophytes contribute to abiotic stress mitigation, secondary metabolite production, and plant adaptation to nutrient-poor environments [2].

The Arabian Peninsula flora provides a unique model to investigate these interactions. In hyper-arid environments, several native and naturalized plants persist where temperature, salinity, and nutrient scarcity would normally hinder vegetation. Although desert microbial ecology has been studied in soils and rhizospheres [3], the endophytic bacterial structure of most desert plants in this region remains unexplored. Only a few investigations have addressed endophyte diversity in extreme arid zones such as the Atacama or Thar deserts [4].

To address this knowledge gap, we examined five co-occurring desert species—*S. papilionacea*, *S. verrucosum*, *O. compressa*, *H. nummularium*, and *C. arvensis*. These plants differ taxonomically but share ecological convergence: they are stress-tolerant, shallow-rooted species that colonize saline, nutrient-poor, or gravelly soils, acting as pioneer vegetation that stabilizes and restores desert ecosystems. Similar convergence among taxonomically diverse hosts has been observed in desert microbiome studies, suggesting that extreme environments select for functionally cohesive microbial communities [5].

*S. papilionacea* (Scrophulariaceae) is a perennial herb adapted to arid mountain slopes and gravel plains, thriving in rocky, nutrient-poor soils and exhibiting strong antioxidant and antifungal properties [6]. *S. verrucosum* (Aizoaceae) is a halophytic succulent with remarkable salinity tolerance, producing osmoprotectants such as ectoine that aid both host and microbial resilience [7]. *O. compressa* (Poaceae) is a desert grass important for sand stabilization and fodder under heat stress [8]. *H. nummularium* (Cistaceae) is a low-growing shrub adapted to dry, calcareous soils, rich in flavonoids and phenolic compounds with antimicrobial potential [9]. *C. arvensis* (Convolvulaceae) is a perennial climber with deep roots conferring drought endurance and soil-binding capacity, known for antioxidant and antibacterial metabolites [10]. Despite their phylogenetic diversity, all five plants share physiological and biochemical traits that mirror their microbial partners’ stress-adaptive roles [11].

This study provides the first comparative analysis of endophytic bacterial communities inhabiting these five desert plants. Using 16S rRNA gene sequencing, we characterized their microbial diversity, taxonomic composition, and predicted metabolic functions to test whether phylogenetically distinct hosts exhibit shared metabolic traits driven by adaptation to aridity. Our work builds upon recent discoveries showing that desert plant microbiomes display functional redundancies and distinct taxa performing similar ecological roles [12]. In this study, we refer to symbiotic plasticity as the capacity of endophytic microbial communities to modulate their functional attributes across phylogenetically distinct host plants, enabling comparable metabolic support under extreme arid conditions. This plasticity reflects the ability of microbes to maintain functional roles despite taxonomic turnover, contributing to host survival in desert environments. This differs from functional redundancy, which implies multiple taxa performing interchangeable functions without regard to environmental pressure, and from ecological filtering, which emphasizes abiotic conditions selecting for taxa capable of surviving stress. Here, functional convergence refers to multiple different microbial taxa exerting the same ecological functions, highlighting the outcome of both processes, microbial communities that are taxonomically variable yet metabolically aligned due to shared environmental constraints. Recent studies support this framework, showing that microbial communities in arid ecosystems exhibit functional resilience despite taxonomic variation [13], that environmental filtering drives alignment of functional traits under harsh abiotic conditions [14], and that desert plants tend to recruit conserved structural and functional endophytic signatures across diverse hosts [15].

## 2. Materials and Methods

**Figure 1:**
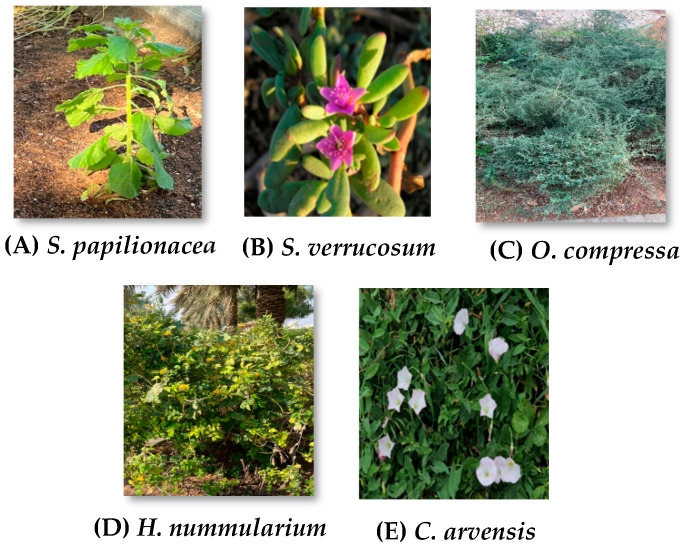
Representative desert plant species were analyzed in this study. Five plants collected from the Al Ain desert region, United Arab Emirates.

Endophytic microbial communities within root tissue were investigated from five desert plant species native or naturalized to the Arabian Peninsula: *S. papilionacea* (A), *S. verrucosum* (B), *O. compressa* (C), *H. nummularium* (D), and *C. arvensis* (E) (Figure 1).

### 2.1. Desert Plant Sample Collection

Sampling was carried out in September 2024 at Al-Ain desert region, United Arab Emirates (coordinates: 24.2075° N, 55.7447° E), an environment characterized by sandy-gravel soils, scarce organic matter, and wide diurnal temperature variation. A total of 25 root samples were processed, each consisting of five pooled plant subsamples collected from different individuals of the same species to capture within-species microbial variability. This pooling strategy aims to reduce individual-level variability and capture a representative snapshot of species-level microbial diversity. Pooling is a common strategy in endophyte studies where the goal is to characterize community-level trends rather than individual host variation. This approach enabled robust alpha- and beta-diversity comparisons across plant species. Immediately after sampling, plant roots were sealed in sterile polyethylene bags, stored in insulated ice containers, and transferred to the laboratory. Samples were maintained at −80 °C until downstream molecular analysis.

### 2.2. Surface Sterilization and DNA Extraction

Prior to DNA isolation, the plant roots underwent surface disinfection to remove epiphytic microorganisms. Segments were immersed in 95% ethanol for 3 min, followed by washing in sterile distilled water, then treated with 3% sodium hypochlorite for 5 min and rinsed again with sterile water. This process was repeated twice. To ensure external decontamination, the final rinse water and tissue imprints were cultured on tryptic soy agar and incubated for three days at 25 °C and 37 °C; the absence of growth confirmed successful sterilization. Genomic DNA was extracted following a modified protocol [16]. Briefly, sterilized tissue (~100 mg) was pulverized in liquid nitrogen using a sterile mortar and pestle and transferred to microcentrifuge tubes containing extraction buffer (200 mM Tris-HCl, pH 7.5, 250 mM NaCl, 25 mM EDTA, and 0.5% SDS). The homogenate was incubated at 65 °C for 10 min to facilitate cell lysis, followed by the addition of chloroform–isoamyl alcohol (24:1) and centrifugation at 12,000× *g* for 10 min to separate phases. The aqueous phase containing DNA was transferred to a fresh tube, and nucleic acids were precipitated with cold isopropanol and washed with 70% ethanol before being resuspended in nuclease-free water. DNA quality was verified using 1% agarose electrophoresis, and concentration was quantified with a NanoDrop™ spectrophotometer (Thermo Fisher Scientific, Waltham, MA, USA).

#### PCR Amplification and 16S rRNA Sequencing

The bacterial 16S rRNA V3–V4 region was amplified using the universal primer pair 341F (5′-CCTACGGGNGGCWGCAG-3′) and 785R (5′-GACTACHVGGGTATCTAATCC-3′) [17]. This pair provides broad bacterial coverage with minimal bias [18,19]. Illumina (San Diego, CA, USA) adapter and linker sequences were added to the primers. Amplification reactions (50 µL) contained 30 ng of template DNA, 0.2 µM of each primer, and Taq polymerase master mix (Sigma ReadyMix™, Coimbatore, India) Taq PCR Reaction Mix (P4600). Thermal cycling included an initial denaturation at 94 °C for 3 min; 30 cycles of 94 °C for 30 s, 56 °C for 45 s, and 72 °C for 45 s; followed by a final 10 min extension at 72 °C. Amplicons were checked on 1.5% agarose gels, purified with the PCR and Gel Cleanup Kit (Enzo Life Sciences, Farmingdale, NY, USA), and quantified using the Quant-iT PicoGreen dsDNA Assay (Seoul, Republic of Korea). Equal DNA quantities were pooled to generate a composite library. Library validation was carried out on an Agilent 2100 Bioanalyzer (Santa Clara, CA, USA), and sequencing was performed by BGI Shenzhen (Shenzhen, China) on an Illumina MiSeq platform (2 × 250 bp paired-end).

### 2.3. Bioinformatic Workflow

Sequencing data were demultiplexed and stripped of adapters and primer sequences. Quality filtering and error correction were executed using DADA2 v1.30.0 [20,21] in R v4.3.2. After denoising, paired reads were merged (mergePairs), and chimeric sequences were eliminated (removeBimeraDenovo). Amplicon Sequence Variants (ASVs) were assigned taxonomies against the Genome Taxonomy Database (GTDB) [22]. Functional inference was carried out with PICRUSt2 v2.3.0-b, which projects likely gene content based on the ASV composition. Predicted functional features were mapped to the KEGG Orthology (KO), Clusters of Orthologous Groups (COG), and MetaCyc databases to identify pathways linked to nutrient metabolism and stress adaptation.

### 2.4. Diversity and Statistical Analyses

Normalization and diversity analyses were conducted using the vegan v2.6-4 [23] and phyloseq v1.46.0 [24] packages within R. Alpha-diversity indices (Chao1, ACE, Shannon, Simpson, and Good’s coverage) were computed using estimate_richness and visualized with ggbetweenstats v0.12.1 [25]. Beta-diversity patterns were explored through Bray–Curtis dissimilarity and displayed via non-metric multidimensional scaling (nMDS) and principal coordinate analysis (PCoA). These ordinations were used to visualize community differentiation among host plants.

## 3. Results

The selected desert hosts’ endophytic bacterial communities were profiled and processed through a standardized bioinformatic workflow to assess microbial diversity and infer predicted functional potential.

### 3.1. Sequencing Output and Rarefaction Analysis

Sequencing of the 25 composite samples yielded approximately 3.4 million paired-end reads. After removing adapters, host DNA, and low-quality fragments, a comparable number of high-quality reads was retained for downstream analysis. De novo clustering at 97% sequence similarity produced operational taxonomic units spanning about 35 phyla, 19 classes, 21 orders, 24 families, and 17 genera, reflecting considerable bacterial diversity across the analyzed endophytic communities.

The rarefaction profiles for all samples (Supplementary Figure S1) showed clear saturation trends, indicating that sequencing depth and sampling effort were adequate to capture the full spectrum of bacterial richness. The curves approached saturation with minimal incremental gain in new ASVs, confirming that most taxa present in the endophytic microbiomes were effectively recovered. Variations in curve elevation were observed among plant species, suggesting differences in community richness, yet all curves stabilized at similar depths, which supports the robustness of the dataset for comparative ecological and functional analyses.

### 3.2. Alpha Diversity of Endophytic Communities

**Figure 2:**
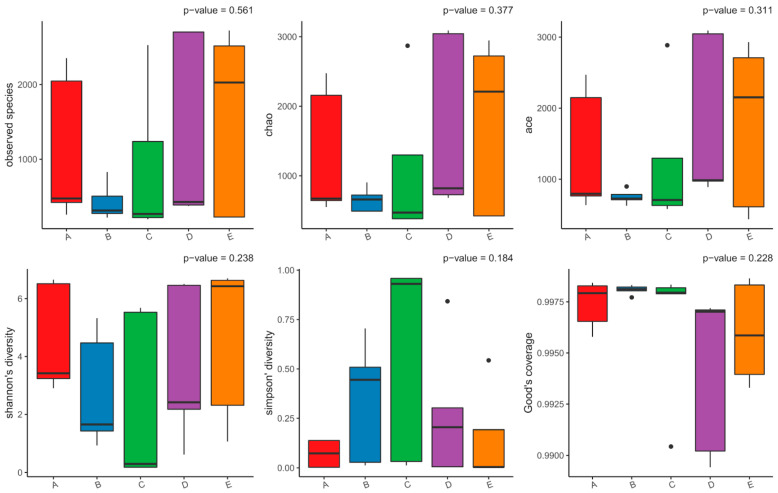
Alpha diversity indices of bacterial endophytes associated with the studied five desert plant species. Boxplots represent six alpha diversity metrics: Observed species, Chao1, ACE, Shannon, Simpson, and Good’s coverage. The consistent values across groups (*p* > 0.05) indicate comparable within-sample diversity and sequencing completeness among plant hosts. A–E represents the 5 studied plant groups.

Alpha diversity provides insight into the richness and evenness of microbial communities within each plant host. In this study, six complementary indices were used to assess within-sample diversity: Observed species, Chao1, ACE, Shannon, Simpson, and Good’s coverage (Figure 2). The richness estimators (Observed, Chao1, and ACE) revealed moderate variation across plants but no statistically significant differences (*p* > 0.05). Samples from *H. nummularium* (D) and *C. arvensis* (E) exhibited slightly higher richness, whereas *S. verrucosum* (B) presented the lowest diversity estimates, suggesting minor host-related variation in bacterial colonization potential. The Shannon and Simpson indices indicated that community evenness remained relatively stable across all hosts, with no evidence of dominance by specific taxa. These balanced profiles suggest that bacterial communities are well-distributed within the endospheric compartments of each species, reflecting comparable ecological pressures across their shared desert habitat. These findings demonstrate that the endophytic bacterial assemblages inhabiting the five desert plants are rich, evenly structured, and statistically comparable, supporting the concept of functional stability and symbiotic equilibrium among hosts adapted to the same extreme environment.

### 3.3. Beta Diversity and Community Dissimilarity

**Figure 3:**
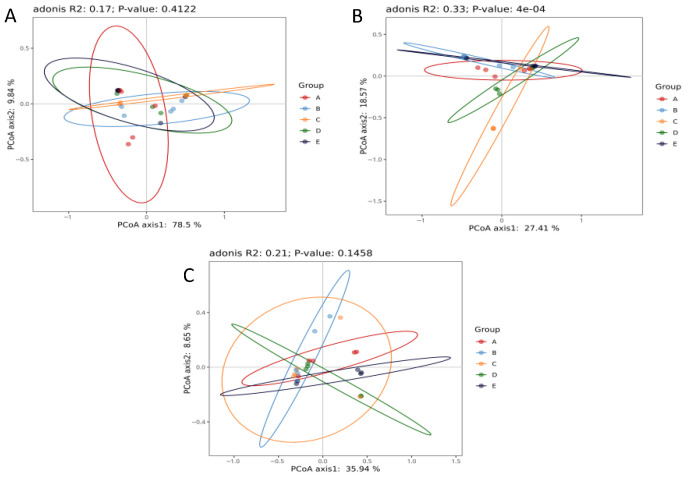
Beta diversity of endophytic bacterial communities visualized using principal coordinate analysis (PCoA). Three distance matrices are used: Bray–Curtis (panel (**A**)), unweighted UniFrac (panel (**B**)), and weighted UniFrac (panel (**C**)). Each point represents one sample; colored ellipses correspond to plant groups (A–E). Bray–Curtis analysis shows significant dissimilarity among hosts (R^2^ = 0.33, *p* = 0.0004), while UniFrac metrics indicate modest, non-significant separation.

Beta diversity was used to assess compositional differences among the endophytic bacterial communities of the five desert plants. Three dissimilarity matrices (Bray–Curtis, unweighted UniFrac, and weighted UniFrac) were calculated and visualized through principal coordinate analysis (PCoA) (Figure 3). The Bray–Curtis dissimilarity metric revealed moderate variation in community composition among the groups (R^2^ = 0.33, *p* = 0.0004), indicating that bacterial assemblages differed markedly across hosts. The clear separation and clustering patterns observed in the PCoA plot suggest that each plant species harbors a partially distinct bacterial community, possibly reflecting subtle host-specific selection pressures within a shared arid ecosystem. By contrast, the weighted UniFrac analysis (R^2^ = 0.17, *p* = 0.4122) showed no significant variation, implying that the dominant and abundant taxa are largely shared across hosts despite differences in their relative abundance. The unweighted UniFrac metric (R^2^ = 0.21, *p* = 0.1458), which accounts for the presence or absence of rare taxa, displayed minor group separation without statistical significance. These results indicate that while taxonomic profiles vary moderately among plant hosts, the overall phylogenetic structure and functional potential remain conserved, consistent with the hypothesis of functional convergence among desert-adapted endophyte communities.

### 3.4. Microbial Composition Across the Studied Plants

**Figure 4:**
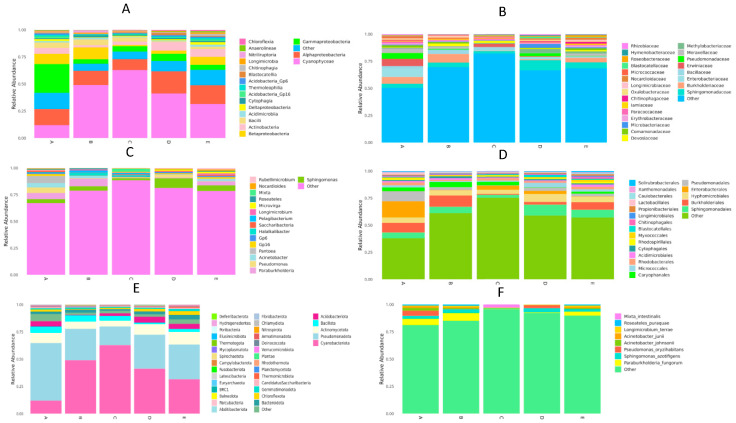
Relative abundance of bacterial taxa at the class and phylum levels across the five plant groups (A–E). Bars represent the mean relative abundance of major bacterial lineages. Panels represent taxonomic levels: (**A**) phylum, (**B**) class, (**C**) order, (**D**) family, (**E**) genus, and (**F**) species.

The comparative taxonomic analysis revealed that all five desert plant hosts harbored diverse but compositionally consistent bacterial communities (Figure 4). Across all five desert plant species, the endophytic microbiota exhibited a coherent taxonomic structure with clear ecological patterns and host-associated distinctions.

At the phylum level, the communities were dominated by *Pseudomonadota*, *Cyanobacteriota*, *Actinomycetota*, and *Bacillota*, with *Acidobacteriota* present at lower abundance. This composition was broadly conserved across hosts, reflecting strong environmental filtering within the hyper-arid landscape. Nonetheless, distinct divergences were noted: *S. papilionacea* (Group A) showed the highest enrichment of *Pseudomonadota*, whereas *O. compressa* (Group C) displayed a pronounced increase in *Cyanobacteriota*. The remaining plants (Groups B, D, and E) exhibited similar overall phylum-level profiles, with minor variability in the relative representation of *Bacteroidota* and *Verrucomicrobiota*. These patterns illustrate how extreme environmental pressures generate a shared microbial backdrop while allowing plant-specific microhabitats to shape finer compositional differences.

At the class level, *Gammaproteobacteria, Alphaproteobacteria*, and *Cyanophyceae* were consistently dominant across all hosts, underscoring the prominence of metabolically flexible and stress-tolerant bacterial lineages in desert endospheres. Yet, each plant species also exhibited distinct taxonomic signatures. Group A was strongly dominated by *Gammaproteobacteria*, while Group B showed notably higher levels of Bacilli and *Cyanophyceae*, likely reflecting the halophytic and succulent traits of *S. verrucosum*. Group C was characterized by a pronounced enrichment of *Cyanophyceae*, consistent with photosynthetically active endophytes in its tissues. Groups D and E displayed more balanced mixtures of *Alphaproteobacteria*, *Actinomycetia* (GTDB class name), and *Cyanophyceae*, suggesting more heterogeneous microhabitats within their tissues. Together, these patterns reveal high-level cross-host similarity overlaying meaningful ecological divergence driven by plant physiology and microenvironmental variability.

Order-level composition further emphasized this interplay between convergence and divergence. Across all hosts, *Sphingomonadales* was the dominant order, accounting for nearly half of total microbial abundance and reflecting its ecological versatility under arid, oligotrophic, and high-radiation conditions. Subdominant orders, including *Burkholderiales* and *Pseudomonadales*, were more enriched in *S. papilionacea* (A), *H. nummularium* (D), and *C. arvensis* (E), aligning with the presence of nitrogen-fixing and siderophore-producing taxa that support nutrient acquisition in desert plants. In contrast, *Enterobacterales* and *Hyphomicrobiales* were more abundant in Groups A and B, suggesting plant-specific recruitment linked to osmotic adjustment and metabolite exchange, particularly in halophytic hosts. Orders such as *Longimicrobiales* and *Rhodospirillales* appeared at moderate but consistent levels across all species, highlighting their ecological roles in oxidative stress protection and organic matter degradation. Group A exhibited the most diverse order-level composition, whereas Group C displayed a more concentrated dominance of *Sphingomonadales*, further demonstrating ecological specialization under extreme water limitation.

Family-level patterns reinforced the presence of a shared functional consortium. All hosts were strongly dominated by *Sphingomonadaceae*, which accounted for more than half of the total abundance and is known for carotenoid biosynthesis, aromatic compound degradation, and stress-protective enzyme production—traits that are highly advantageous in arid ecosystems. Subdominant families, including *Burkholderiaceae* and *Pseudomonadaceae*, were notably enriched in Groups A and D, consistent with enhanced nitrogen fixation, phosphate solubilization, and antimicrobial metabolite production in these hosts. *Enterobacteriaceae* and *Bacillaceae* were more prominent in *S. verrucosum* (Group B), reflecting adaptations to saline or osmotic stress. Several additional families, such as *Comamonadaceae*, *Rhizobiaceae*, *Microbacteriaceae*, *Hymenobacteraceae*, and *Nocardiaceae,* were present at lower but persistent levels across hosts, contributing specialized ecological functions such as radiation resistance, biofilm formation, and osmoprotectant synthesis. The combination of widespread core families and host-specific secondary families illustrates how desert plants maintain a stable yet flexible microbial framework.

Genus-level profiles revealed *Sphingomonas* as the dominant genus in all plants, comprising over half of the relative abundance and appearing as a keystone lineage across the five hosts. Its broad ecological tolerance and metabolic versatility make it highly suited to colonizing multiple plant species under desert conditions. Subdominant genera, including *Paraburkholderia*, *Pseudomonas*, and *Acinetobacter*, were consistently represented across hosts, forming a shared functional guild associated with nutrient cycling, nitrogen fixation, siderophore secretion, and organic matter turnover. Host-specific patterns included measurable levels of *Rubellimicrobium* and *Roseateles* in Groups A and B, indicating selection for taxa adapted to nutrient-poor or high-radiation microenvironments. Similarly, *Longimicrobium* and *Microvirga* were more prominent in Groups D and E, potentially contributing osmoprotective metabolites and amino-acid-related functions. Minor genera such as *Mixta*, *Nocardioides*, and *Pelagibacterium* further enriched the functional diversity of the communities.

Species-level analysis suggests a shared adaptive core along with meaningful host-specific variation. *Acinetobacter johnsonii* was the most abundant species in all hosts, representing a universal endophytic component with recognized roles in desiccation tolerance, carbon substrate utilization, and extracellular enzyme production. *Paraburkholderia fungorum* was the second most widespread species, contributing to nitrogen fixation, phosphate solubilization, and phytohormone synthesis that support plant resilience under arid conditions. Additional taxa, including *Pseudomonas oryzihabitans*, *Sphingomonas azotifigens*, and *Acinetobacter junii*, showed moderate but variable abundances across hosts, while *Microbacterium intestinalis* appeared uniquely in *S. verrucosum* (Group B), suggesting a halophyte-specific microenvironmental effect. The overall richness gradient (A ≈ E > D > B > C) likely reflects differences in plant tissue architecture, root system diversity, and microhabitat heterogeneity.

Taken together, these taxonomic profiles demonstrate that the five plant species share a coherent microbial foundation shaped by desert environmental filtering, while also exhibiting ecologically meaningful divergences driven by host traits and microhabitat conditions. The dominance of metabolically flexible and stress-tolerant taxa, combined with subtle host-specific compositional shifts, suggests a pattern of functional convergence across phylogenetically diverse desert plants and highlights the ecological plasticity of their associated microbiomes.

### 3.5. Functional Prediction and Metabolic Pathway Analysis

**Figure 5:**
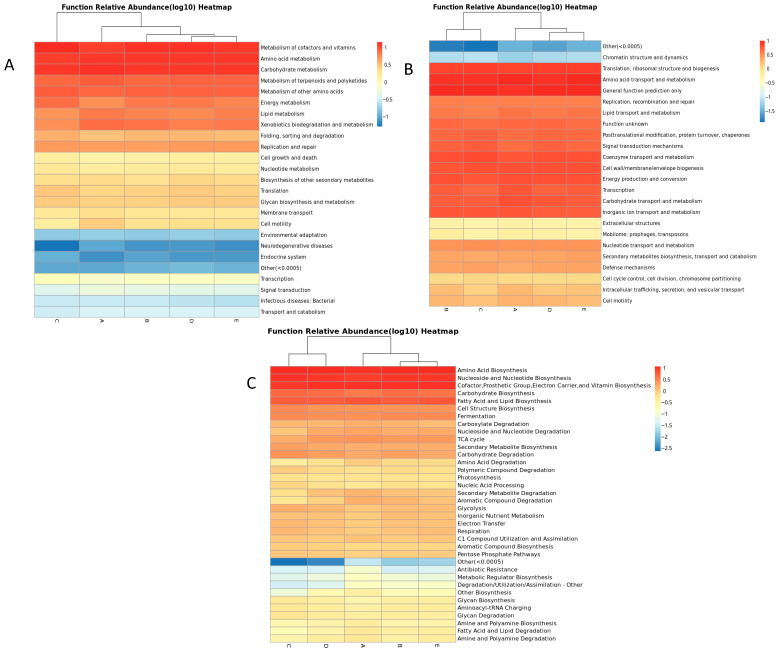
Predicted functional profiles of endophytic bacterial communities based on PICRUSt2 analysis. Heatmaps represent the relative abundance (log_10_ scale) of predicted functions derived from (**A**) COG/KOG, (**B**) MetaCyc, and (**C**) KEGG Orthology (KO) databases.

The predicted functional potential of the bacterial endophytes was evaluated using the PICRUSt2 pipeline, which mapped gene functions to three major databases: Clusters of Orthologous Groups (COG/KOG), Kyoto Encyclopedia of Genes and Genomes (KO), and MetaCyc (Figure 5). Across all five plant groups, the PICRUSt2-based heatmaps revealed a consistent distribution of predicted functional categories, indicating that the endophytic communities share a broadly conserved metabolic profile. This stability reflects the high degree of predicted functional adaptability of desert microbiomes to nutrient limitation and environmental stress. In the COG/KOG analysis, predicted functions associated with amino acid, carbohydrate, lipid, and coenzyme metabolism were the most abundant, followed by energy production and conversion, protein modification and turnover, and post-translational processes. Moderate enrichment was observed for categories related to secondary metabolite biosynthesis, defense mechanisms, and nucleotide transport and metabolism, whereas cell motility and signal transduction were among the least represented.

The MetaCyc-based functional reconstruction showed a predicted dominant activity in amino acid, nucleotide, vitamin, and lipid biosynthesis, with additional contributions from carbohydrate and aromatic compound degradation pathways. These results emphasize the communities’ metabolic versatility and their capacity for nutrient recycling within the plant endosphere. Of note, expression of antibiotic-resistance-related pathways remained consistently low across all groups. The KO-based predictions confirmed similar enrichment patterns, highlighting amino acid, carbohydrate, and lipid metabolism, along with the biosynthesis of terpenoids, polyketides, and secondary metabolites. Predicted functions related to glycan biosynthesis and energy metabolism were also widely shared among the five hosts, suggesting a conserved metabolic foundation across phylogenetically distinct endophytic assemblages. These results indicate that the desert plant endophytes maintain a metabolically cohesive and functionally resilient core, dominated by pathways essential for biosynthesis, nutrient cycling, and stress adaptation. These patterns indicate a common set of stress-associated microbial predictive functions among the five species examined, although broader sampling is needed to determine the extent to which such patterns generalize across desert flora.

## 4. Discussion

This study provides the first comparative overview of the endophytic bacterial communities inhabiting the studied desert plant species native or naturalized to the Arabian Peninsula. By integrating 16S rRNA amplicon sequencing and predictive functional analyses, we reveal that despite taxonomic variability among hosts, the overall microbial architecture remains compositionally stable and functionally cohesive. Such structural convergence suggests that desert plants may recruit and maintain a conserved microbial consortium through ecological filtering and metabolic compatibility, resulting in functional convergence and symbiotic plasticity under extreme environmental constraints [12].

The five hosts represent diverse ecological niches spanning halophytic to xerophytic habitats. Yet across all, a consistent microbial signature dominated by *Pseudomonadota*, *Actinomycetota*, and *Bacillota* was observed, with *Sphingomonas*, *Paraburkholderia*, *Pseudomonas*, and *Acinetobacter* forming the core taxa. This cross-host uniformity highlights the desert microbiome’s reliance on metabolically flexible lineages capable of osmoprotection, nutrient mobilization, and redox balance. *Sphingomonas* and *Sphingomonadaceae*, the most dominant taxa, are well known for their degradation of aromatic compounds and carotenoid biosynthesis, both essential for UV protection and oxidative stress mitigation [26]. The recurrence of *Burkholderiaceae* and *Pseudomonadaceae* underscores their ecological roles in nitrogen fixation, phosphate solubilization, and production of siderophores and phytohormones that enhance drought tolerance [27]. Together, these findings indicate that desert endophytes form a core functional guild optimized for survival under aridity and resource scarcity.

### 4.1. Ecological Cohesion and Functional Convergence

Although relative abundances varied slightly among hosts, the observed alpha- and beta-diversity patterns reflect a stable equilibrium between microbial diversity and host specificity. Such a balance supports the view that arid ecosystems favor microbial communities exhibiting functional redundancy rather than taxonomic diversity, ensuring essential ecosystem processes persist despite compositional shifts [28]. The co-dominance of *Actinomycetota* and *Cyanobacteriota* illustrates complementary ecological functions: the former degrades recalcitrant organic matter and contributes to nitrogen cycling, while the latter stabilizes rhizosphere microenvironments through photosynthetic carbon input and exopolysaccharide production [29]. The persistence of radiation-resistant lineages such as Deinococcota and thermotolerant *Chloroflexota* reinforces the existence of a desert-adapted microbial blueprint, a conserved set of taxa and functions sustaining microbial–plant homeostasis under high radiation, salinity, and temperature [15].

Predicted functional inference via PICRUSt2 may further support this ecological cohesion. Core pathways, including amino acid, carbohydrate, and lipid metabolism, together with post-translational modification and cofactor biosynthesis, were consistently enriched, demonstrating a shared metabolic backbone across hosts. These predicted functions may reflect the need for flexible energy generation and biomolecule synthesis under nutrient limitation and oscillating hydration regimes [30]. Elevated expression of amino acid and vitamin biosynthetic routes implies a role in maintaining host nutritional balance, consistent with mutualistic rather than competitive interactions [2]. These results portray a microbiome that is not only compositionally resilient but also functionally synchronized with plant metabolic needs. We acknowledge that PICRUSt2 provides predictive rather than direct functional measurements; because 16S rRNA–based inference relies on reference genomes, functional profiles for novel or poorly characterized desert taxa may be underestimated. Thus, the functional predictions presented here should be interpreted as indicative trends rather than definitive metabolic capabilities.

### 4.2. Linking Plant and Microbial Functional Plasticity

Desert plants such as *S. papilionacea*, *S. verrucosum*, *O. compressa*, *H. nummularium*, and *C. arvensis* are characterized by remarkable physiological plasticity, adjusting phenology, osmolyte production, and resource allocation to survive fluctuating aridity (Gairola et al., 2021) [31]. Our data indicate that this adaptability is mirrored by their microbial symbionts, which exhibit metabolic and symbiotic flexibility across hosts. Dominant *Alphaproteobacteria*, particularly *Sphingomonas* and *Rhizobium*-related taxa, are capable of auxin and ACC-deaminase synthesis, directly influencing plant growth and stress signaling. *Actinomycetota* (class *Actinomycetia*) and *Cyanobacteriota* complement these functions by producing osmoprotectants, extracellular polysaccharides, and antioxidant enzymes that extend the host’s physiological tolerance [31]. This dual adaptability forms a coevolutionary feedback loop: plastic plant traits create niche heterogeneity that selects for metabolically versatile microbes, which in turn stabilize plant function under stress. Similar host–microbe modularity has been documented in desert holobionts, where endophytes mediate aquaporin regulation and nutrient foraging, enhancing survival across microhabitats [32].

The desert hosts investigated here possess physiological adaptations such as shallow or fibrous roots, enhanced osmolyte production, UV-protective secondary metabolites, and efficient water-retention mechanisms. These features generate selective microhabitats that favor endophytic partners capable of complementary functions, including nitrogen fixation, phosphate solubilization, oxidative stress mitigation, osmoprotectant and carotenoid production, and extracellular polymer synthesis.

### 4.3. The Desert Microbial Blueprint and Biotechnological Potential

Together, these findings position desert endophytes as components of a predictable, functionally convergent microbiome, a blueprint of ecological resilience built on stress-tolerant, metabolically redundant taxa. This convergence likely arises from long-term environmental filtering and co-adaptive host selection, yielding microbial assemblages that secure nutrient cycling, oxidative stress defense, and osmotic balance in extreme conditions [2]. The parallel between plant functional plasticity and microbial metabolic flexibility underscores the desert holobiont as a unified adaptive system, where resilience emerges from mutual reinforcement of physiological and metabolic plasticity [1].

Beyond ecological insight, this conserved desert microbiome provides a foundation for applied biotechnology. Isolating key taxa such as *Sphingomonas*, *Pseudomonas*, and *Paraburkholderia* could inform the development of microbial bioinoculants that enhance drought resistance, carbon sequestration, and soil fertility in degraded lands [33]. Understanding these metabolic networks also supports the design of sustainable crop systems aligned with the United Nations’ climate adaptation goals.

## 5. Conclusions

In conclusion, our findings provide initial evidence of functionally convergent endophytic traits across co-occurring host plants in a hyper-arid environment. Although further multi-sites as well as seasonal sampling will be significant for further host-specific pattern confirmation. Despite their phylogenetic diversity, the five desert plant species examined harbor microbiomes dominated by metabolically flexible and stress-tolerant taxa that likely contribute to plant resilience under extreme abiotic pressures [15,34]. In our study, the functional analysis was conducted based on PICRUSt2 predictions derived from 16S RNA profiles, which represent the metabolic potential of the microbial community rather than confirm it; thus, future multi-omics analyses are required to validate our predicted functional statements. Integration of metagenomics and metabolomics, along with cultivation-based assays, will be essential to unravel the active metabolites and signaling pathways that underpin this resilience in desert ecosystems.

Beyond revealing shared microbial features, our study provides a broader integrative synthesis linking plant traits, microbial functional capacities, and environmental filtering into a unified conceptual framework. The recurrent enrichment of key microbial genera such as *Sphingomonas*, *Paraburkholderia*, *Pseudomonas*, and *Acinetobacter* illustrates how environmental filtering promotes a shared microbial backbone, while plant-specific chemical environments fine-tune community composition through selective recruitment [15,33]. Our study expands the foundation for future work exploring how microbial partnerships sustain plant survival in some of Earth’s most inhospitable environments.

Our conclusions are therefore based on functional inference derived from metagenomic datasets rather than direct physiological or experimental validation. While the observed conservation of functional gene repertoires across taxonomically distinct microbial assemblages suggests potential functional redundancy, the extent to which these communities maintain stable metabolic activities and confer consistent stress-mitigating effects on their host plants remains to be experimentally verified. Future studies integrating microbial isolation, synthetic community reconstruction, and plant inoculation assays under controlled stress conditions will be necessary to directly test these functional relationships.

## Data Availability

All sequences have been deposited into SRA with the following accession numbers: SAMN52920307, SAMN52920308, SAMN52920309, SAMN52920310, SAMN52920311 (https://www.ncbi.nlm.nih.gov/biosample/52920307 (accessed on 21 October 2025), https://www.ncbi.nlm.nih.gov/biosample/52920308 (accessed on 21 October 2025), https://www.ncbi.nlm.nih.gov/biosample/52920309 (accessed on 21 October 2025), https://www.ncbi.nlm.nih.gov/biosample/52920310 (accessed on 21 October 2025), https://www.ncbi.nlm.nih.gov/biosample/52920311 (accessed on 21 October 2025)).

## Supplementary Materials

The following supporting information can be downloaded at: https://www.mdpi.com/article/10.3390/microorganisms14040836/s1, Figure S1: Rarefaction curves of bacterial endophytic communities across the five desert plant groups (A–E). Each curve represents the number of observed Amplicon Sequence Variants (ASVs) as a function of sequencing depth. The asymptotic trend of all curves indicates sufficient sequencing coverage and sampling depth for reliable diversity analysis.
