# Supplementary figures and images for: Shared Microbial Blueprints Underlying Symbiotic Plasticity in Desert Plant Endophytes
Source: Microorganisms. 2026 Apr 7;14(4):836. doi: 10.3390/microorganisms14040836 (PMC13118546; doi:10.3390/microorganisms14040836)

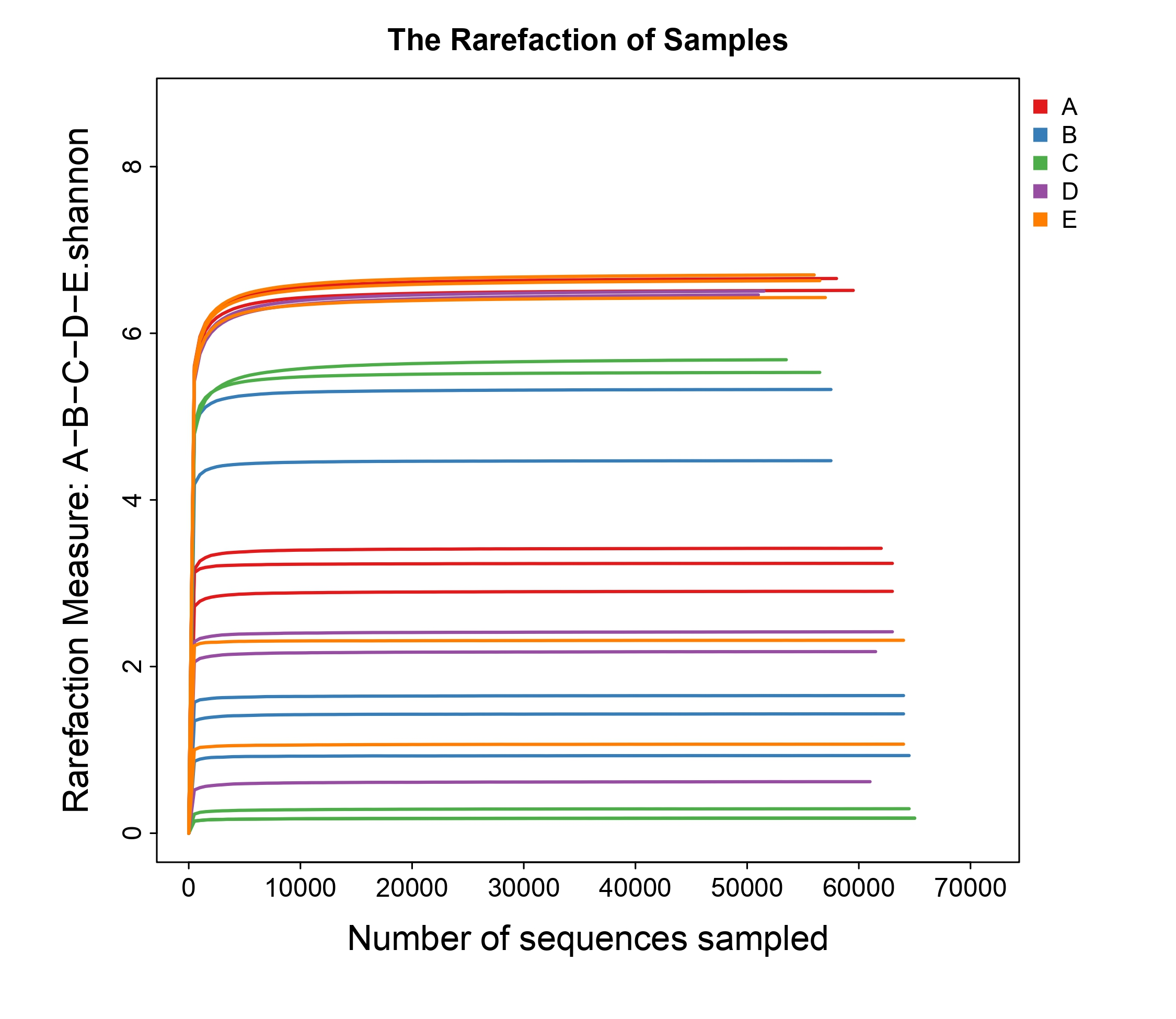

Supplement: Supplementary file 1 [file microorganisms-14-00836-s001.zip › microorganisms-4187551-supplementary.tiff]
